# Protocol to generate traceable CAR T cells for syngeneic mouse cancer models

**DOI:** 10.1016/j.xpro.2024.102898

**Published:** 2024-02-16

**Authors:** Duo Zhang, Elisavet Krimitza, Katherine Han, Ruiying Su, David J. Xu, Jaiden R. Xu, Yanqing Gong, Yi Fan

**Affiliations:** 1 Department of Radiation Oncology, University of Pennsylvania, Philadelphia, PA 19104, USA; 2 Department of Pathology, University of Pennsylvania, Philadelphia, PA 19104, USA; 3 Department of Medicine, University of Pennsylvania, Philadelphia, PA 19104, USA

**Keywords:** Cell culture, Cell isolation, Flow Cytometry, Cancer, Immunology, Model Organisms

## Abstract

The efficacy of chimeric antigen receptor (CAR) T cell immunotherapy is limited by insufficient infiltration and activation of T cells due to the immunosuppressive tumor microenvironment. Preclinical studies with optimized mouse CAR T cells in immunocompetent mouse cancer models will help define the mechanisms underlying immunotherapy resistance. Here, we present a protocol for preparing mouse T cells and generating CAR T cells. We then detail procedures for testing their therapeutic efficacy and tracking them in a syngeneic mouse glioma model.

For complete details on the use and execution of this protocol, please refer to Zhang et al.^1^

## Highlights

• Generation of mouse CAR T cells using retrovirus
• *In vitro* testing cytotoxicity of CAR T cells
• Preclinical testing of CAR T cells with a syngeneic mouse glioma model
• *In vivo* bioluminescence tracking of CAR T cells in mice

## Abstract (editor highlights)

Publisher’s note: Undertaking any experimental protocol requires adherence to local institutional guidelines for laboratory safety and ethics.

## Graphical abstract

**Figure:**
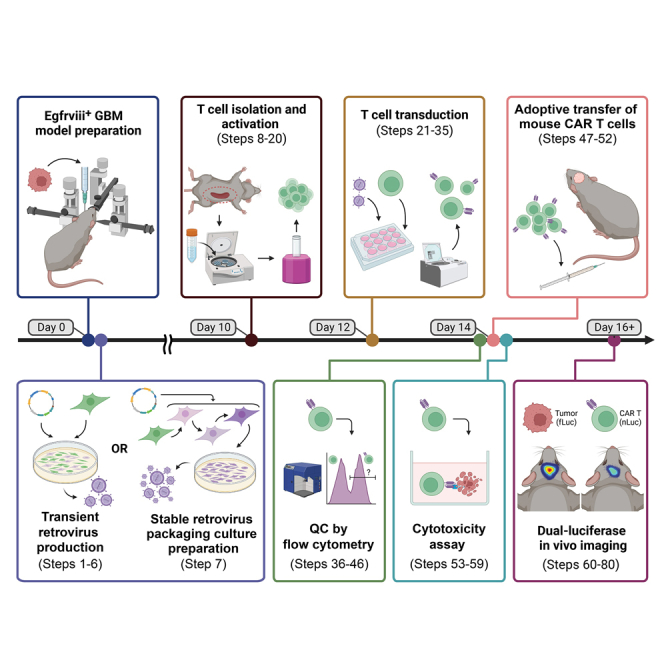

## Before you begin

**Figure 1:**
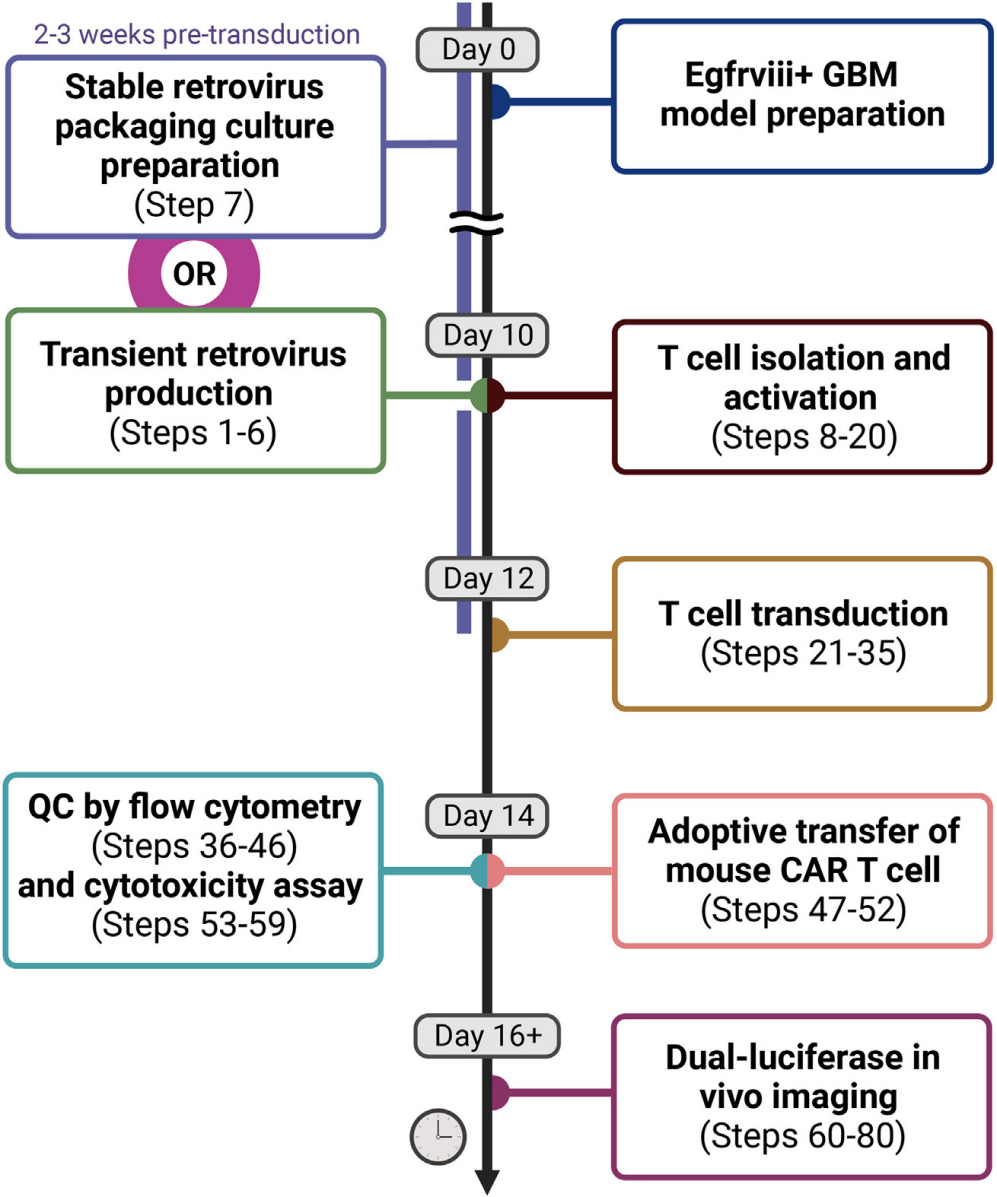
Timeline of the experimental procedure

***Note:*** This protocol outlines the procedure of expressing a chimeric antigen receptor (CAR) using a Moloney murine leukemia (MMLV) retroviral vector, integrated with a nano-luciferase (nLuc) and fluorescent protein tracing system. This system facilitates the tracking of CAR T cell infiltration and localization in syngeneic mouse cancer models through a bioluminescence-based *in vivo* imaging system. The specific CAR construct employed in this protocol incorporates the 139 scFv,^2^^,^^3^ targeting the Egfrviii—a glioblastoma (GBM)-associated variant of the epidermal growth factor receptor (EGFR). Following the protocol, we administered CAR T immunotherapy to mice with syngeneic GL261 GBM tumors expressing murine Egfrviii. This approach holds promise for various CAR-antigen combinations, animal models, and transgene carrier vectors. The whole procedure will take about 2 weeks to complete (Figure 1).

### Institutional permissions

All experimental procedures involving live animals were conducted in strict accordance with the guidelines and regulations set forth by the University of Pennsylvania’s Institutional Animal Care and Use Committee (IACUC). Researchers and institutions intending to replicate or adapt this protocol must ensure they obtain the necessary permissions and approvals from their respective institutional committees. It is imperative to adhere to local, state, and national regulations and guidelines pertaining to animal care, biosafety, and research ethics.

### Preparation of traceable CAR vector

**Timing: 1–2 weeks**

**Figure 2:**
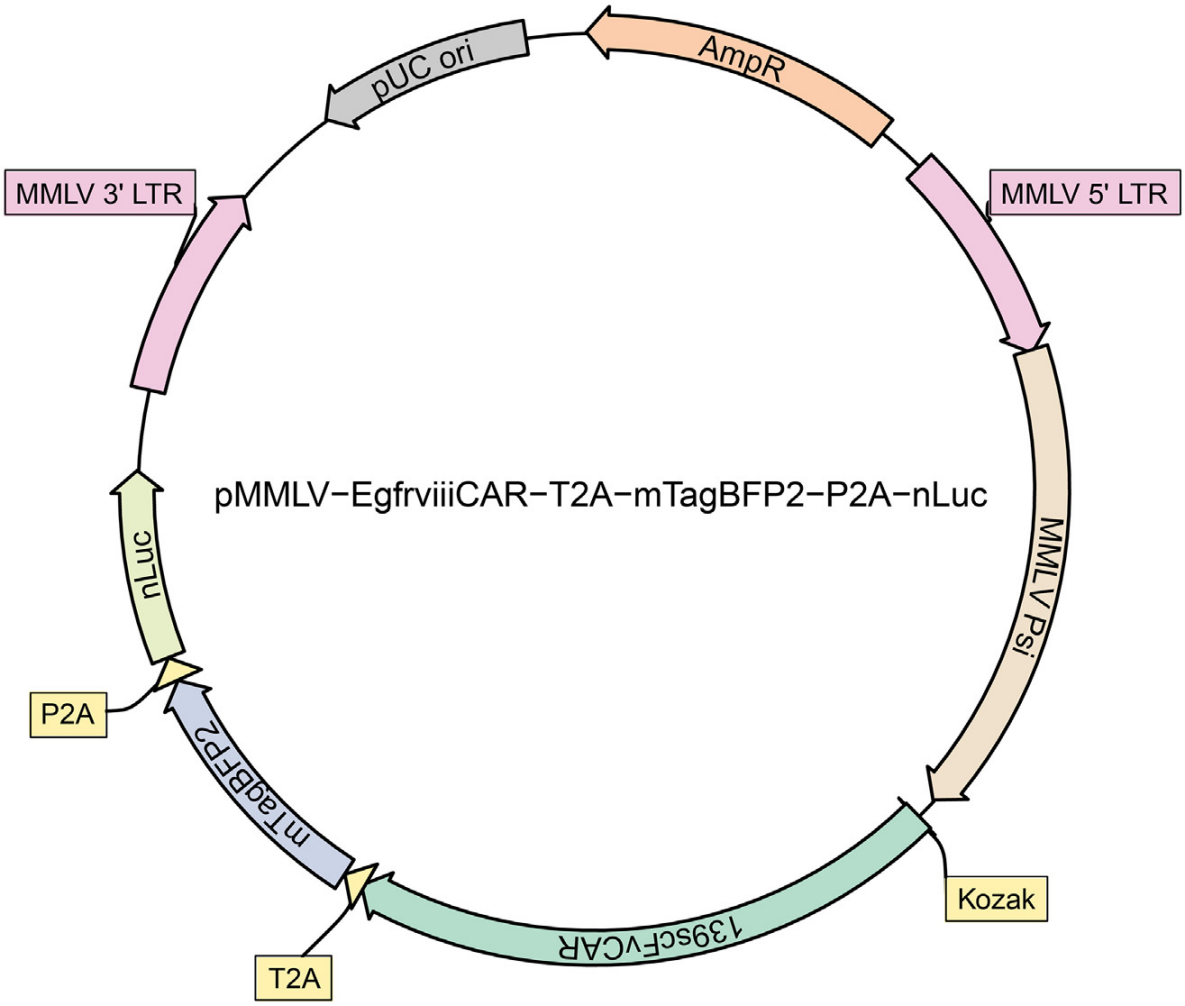
Schematic representation of the pMMLV-139scFvCAR-T2A-mTagBFP2-P2A-nLuc plasmid The inserted sequence encodes for the 139scFv chimeric antigen receptor (CAR), the monomeric blue fluorescent protein (mTagBFP2), and the nano-luciferase (nLuc) reporter. Rendered by R package plasmapR.^7^

1. Synthesize the polycistronic traceable CAR vector that expresses the CAR of interest, a fluorescent reporter, and a bioluminescent reporter (Figure 2). The three products will share a single open reading frame and be separated by 2A peptide sequences for efficient and stoichiometric production of discrete protein products.

***Note:*** For the vector (pMMLV-Egfrviii-CAR-T2A-mTagBFP2-P2A-nLuc, (Figure 2) described here, we chose mTagBFP2 as the fluorescent reporter and nLuc as the bioluminescent reporter. For the fluorescent reporter, proteins with a different excitation/emission spectrum or quantum yield may be used based on your experimental needs. We chose nLuc for its robust emission intensity,^4^ which allows optimal sensitivity for visualizing rare cell populations in the tumor microenvironment. Alternatively, luciferases with similar characteristics or kinetics, such as Antares^5^ or teLuc,^6^ may be used with their optimal substrates, but have not been tested by us.

### Preparation of syngeneic Egfrviii+ glioblastoma model

**Timing: 1–2 weeks**

***Note:*** Detailed steps for the generation of the GBM animal model have been described previously.^1^^,^^3^^,^^8^ We provide a brief overview of the steps in this section.

2. Administer an orthotopic and stereotactic injection to approximately 8-week-old recipient mice with 1 × 10^5^ Egfrviii^+^ GL261 mouse GBM tumor cells, resuspended in PBS to a final volume of 1 μL.
3. 10 days post-injection, confirm and monitor tumor growth via bioluminescence using an *in vivo* imaging system (IVIS) following a retro-orbital injection of 50 μL of D-luciferin solution per mouse.

**CRITICAL:** Careful planning is required to obtain ready-to-inject CAR T cells for the optimal treatment window. Animal model preparation is often performed in parallel with CAR T cell transduction and preparation, which will be described later in this protocol. Target an injection time frame of 12–15 days following the tumor cell injection.

## Key resources table

**Table:** 

REAGENT or RESOURCE	SOURCE	IDENTIFIER
**Antibodies**		

Purified anti-mouse CD3ε antibody dilution: 1:100 (for T cell culture)	BioLegend	Cat# 100301, RRID: AB_312666
Purified anti-mouse CD28 antibody dilution: 1:100 (for T cell culture)	BioLegend	Cat# 102101, RRID: AB_312867
Alexa Fluor 647 AffiniPure Goat anti-human IgG, F(ab')₂ fragment specific dilution: 1:200 (flow cytometry)	Jackson ImmunoResearch	Cat# 109-605-006, RRID: AB_2337881

**Chemicals, peptides, and recombinant proteins**		

D-luciferin, sodium salt	GoldBio	Cat# LUCNA-5G
HEPES (1 M)	Gibco	Cat# 15630130
Recombinant murine IL-2	PeproTech	Cat# 212-12
RetroNectin	Takara	Cat# T100A
EDTA (0.5 M)	Invitrogen	Cat# AM9260G
Sodium pyruvate (100 mM)	Gibco	Cat# 11360070
NEAA (100×)	Gibco	Cat# 11140050
2-Mercaptoethanol (50 mM)	Gibco	Cat# 31350010
Puromycin dihydrochloride	Gibco	Cat# A1113803
Blasticidin S HCl	Gibco	Cat# A1113903
Furimazine	TargetMol	Cat# T15359
Furimazine	Fisher Scientific	Cat# NC2103302

**Critical commercial assays**		

Lipofectamine 3000 transfection reagent	Thermo Scientific	Cat# L3000001
EasySep Mouse T Cell Isolation Kit	STEMCELL Technologies	Cat# 19851

**Experimental models: Cell lines**		

Egfrviii^+^ GL261 mouse glioma cells	Zhang et al.^1^	N/A
Platinum-ECO cells	Cell Biolabs	Cat# RV-101, RRID: CVCL_B488

**Experimental models: Organisms/strains**		

C57BL/6J mice (wild-type, 6–8 weeks old, half male and half female)	Jackson Laboratory	RRID: IMSR_JAX:000664

**Recombinant DNA**		

pMMLV-139scFvCAR-T2A-mTagBFP2-P2A-nLuc plasmid	This paper; by Vectorbuilder	Custom order

**Software and algorithms**		

FlowJo v10	TreeStar	https://www.flowjo.com/
Prism 9	GraphPad	https://www.graphpad.com/scientificsoftware/prism/
Living Image v4.8	PerkinElmer	https://www.perkinelmer.com/product/spectrum-200-living-image-v4series-1-128113

**Other**		

PBS, 1× without calcium and magnesium	Corning	Cat# 21-040-CM
DMEM w/ L-glutamine, 4.5 g/L glucose and sodium pyruvate	Gibco	Cat# 11995065
IMDM w/ L-glutamine and 25 mM HEPES	Gibco	Cat# 12440053
FBS	Sigma	Cat# F4135
Antibiotic-Antimycotic (100×)	Gibco	Cat# 15240062
Opti-MEM reduced serum medium (1×)	Gibco	Cat# 31985062
Millex PVDF syringe filter	Sigma-Aldrich	Cat# SLHVR33RS
Steriflip-HV sterile centrifuge tube top filter unit	Sigma-Aldrich	Cat# SE1M003M00
70 μm cell strainer	Corning	Cat# 352350

## Materials and equipment

• **D10 medium.**

**Table:** 

Reagent	Final concentration	Amount
DMEM w/ L-Glutamine, 4.5 g/L Glucose and Sodium Pyruvate	N/A	430 mL
FBS	10% (vol %)	50 mL
HEPES (1 M)	20 mM	10 mL
NEAA (100×)	1% (vol %)	5 mL
Antibiotic-Antimycotic (100×)	1% (vol %)	5 mL
**Total**	**N/A**	**500 mL**

***Note:*** store at 4°C for up to 2 months.

• **T cell medium (TCM).**

**Table:** 

Reagent	Final concentration	Amount
IMDM w/ L-Glutamine and 25 mM HEPES	N/A	434 mL
FBS	10% (vol %)	50 mL
Sodium pyruvate (100 mM)	1 Mm	5 mL
NEAA (100×)	1% (vol %)	5 mL
Antibiotic antimycotic solution (100 x)	1% (vol %)	5 mL
2-Mercaptoethanol (50 mM)	0.05 mM	0.5 mL
rmIL-2 stock solution (5 × 10^4^ U/mL)	50 U/mL	0.5 mL
**Total**	**N/A**	**500 mL**

***Note:*** store at 4°C for up to 2 weeks.

• **rmIL-2 stock solution**: prepare 5 × 10^4^ U/mL stock of recombinant murine IL-2 solution according to the data sheet and the lot-specific certificate of analysis. Aliquot to prevent freeze-thaw cycling.

***Note:*** store at −20°C for up to 12 months.

• **MACS buffer**: add 5 mL FBS (1%) and 2 mL of 0.5 M EDTA (2 mM) to 493 mL PBS.

***Note:*** store at 4°C for up to 2 months.

• **D-luciferin solution**: solve 1 g of D-luciferin sodium salt in 25 mL sterile PBS (125 mM). Filter with a 0.45 μm filter and aliquot.

***Note:*** store at −20°C for up to 6 months.

• **Furimazine 20**× **stock solution**: solve 1 mg of furimazine in 0.5 mL of DMSO (5.24 mM). Filter with a 0.45 μm filter and aliquot.

***Note:*** store at −20°C for up to 6 months.

## Step-by-step method details

### Ecotropic retrovirus packaging by Platinum-ECO cells

**Timing: 3 days for virus production by transient transfection; ∼2–3 weeks for stable virus-packaging cell culture production**

**Figure 3:**
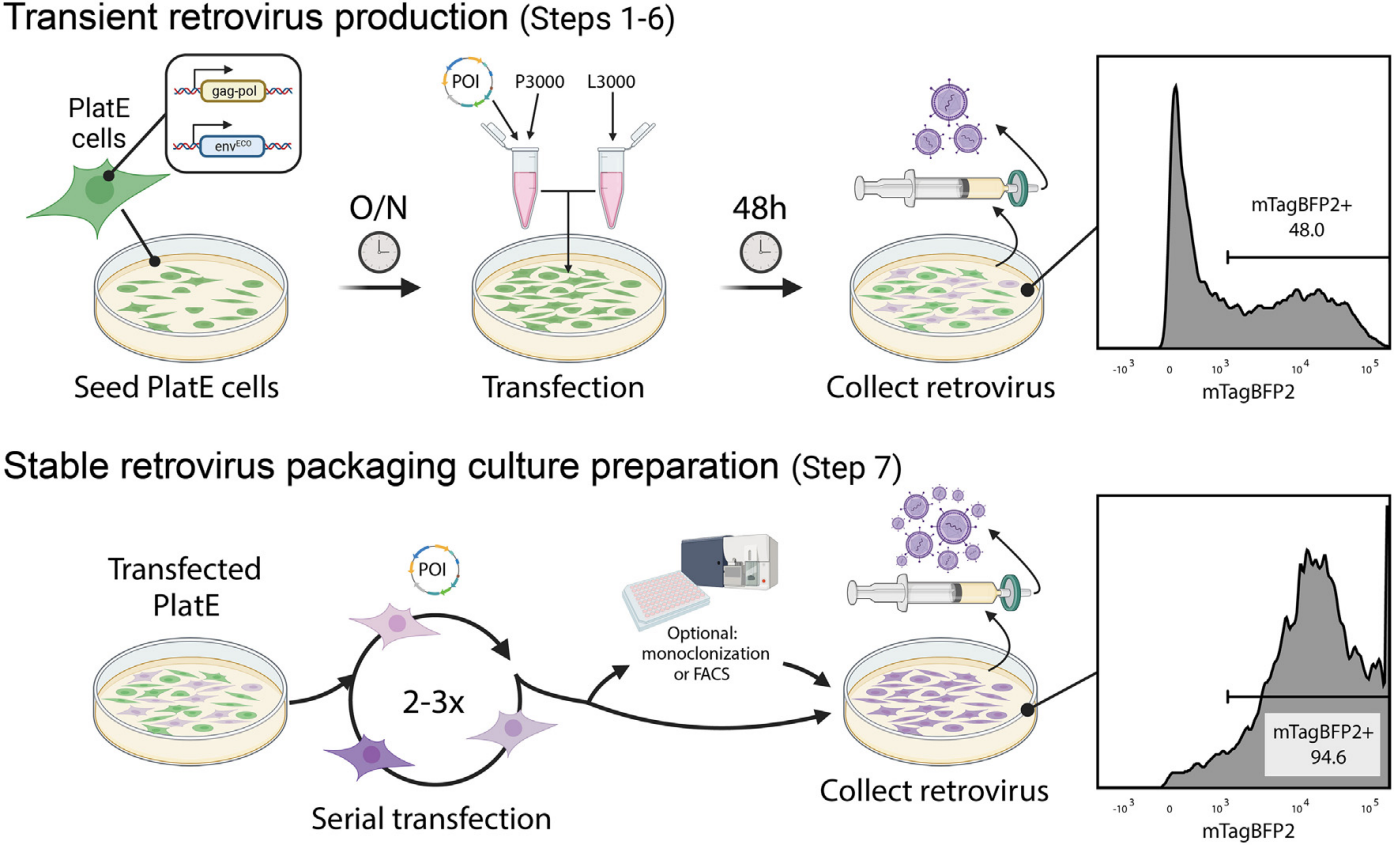
Overview of the procedures for transient and stable retrovirus production For transient production (Steps 1-6): PlatE cells are seeded and subsequently transfected with the plasmid of interest (POI). After 48 h, the produced retrovirus is collected, and the transfection efficiency is analyzed by flow cytometry on mTagBFP2 expression. For stable retrovirus packaging culture preparation (Step 7): Transfected PlatE cells undergo another 2-3 rounds of serial transfection, with an optional purification step for monoclonalization or Fluorescence-Activated Cell Sorting (FACS). The retrovirus is then collected. Viral genome integration is assessed by mTagBFP2 expression again using flow cytometry, showing a significantly increased expression.

This section details the introduction of the pMMLV traceable CAR construct into Platinum-ECO cells (PlatE) to produce ecotropic gamma-retrovirus for subsequent gene transfer into mouse T cells. PlatE cells express the viral structural genes gag-pol and env, permitting the stable production of high-titer retroviruses without requiring a helper plasmid. However, the enlarged viral genome size, due to the inclusion of reporter genes, may impact the viral titer during transient virus production, potentially resulting in a reduced transduction rate. To address this issue, a stable virus-packaging PlatE culture with a high copy number of the integrated viral genome can be established through serial transfections (Figure 3).

1. Thaw PlatE cells (3–5 × 10^6^ cells per vial, passages 2–6 ideally) in 37°C water bath. Maintain the cell culture in D10 media with 10 μg/mL blasticidin and 1 μg/mL puromycin for a continuous selection of gag-pol^+^ and env^+^ cells.
2. Seed approximately 5 × 10^6^ PlatE cells in a 10 cm TC-treated dish for each vector of interest. Incubate overnight (12–16 h) in D10 media at 37°C with 5% CO_2_.

***Note:*** Avoid uneven distribution or cell aggregation as it can impact transfection efficiency. To ensure a single-cell suspension during seeding, consider filtering the PlatE cells through a 70 μm cell strainer.

3. On the following day, ensure the cells have reached an optimal confluency of 70–80% before proceeding with the transfection. Troubleshooting problem 1.
4. Use the Lipofectamine 3000 system to prepare the transfection reaction and transfect the PlatE cells:
  a. For each vector, combine 0.5 mL of Opti-MEM media and 40 μL of Lipofectamine 3000 reagent in a 1.5 mL Eppendorf tube. Mix well by tapping.
  b. In another 1.5 mL Eppendorf tube, for each vector, add 0.5 mL of Opti-MEM media, 20 μg of each desired vector (for this protocol, pMMLV-Egfrviii-CAR-T2A-mTagBFP2-P2A-nLuc is used), and 40 μL of P3000 reagent. Begin by adding the plasmid DNA to ensure it dissolves completely in Opti-MEM, followed by the P3000. Mix well.
  c. Gently pipette to combine the contents of the two Eppendorf tubes and allow them to incubate at room temperature (20°C–22°C) for 15 min. Do not vortex.
  d. Add the resulting mixture dropwise to the dish containing the PlatE cells in 10 mL culture medium, gently swirling the dish to ensure even distribution. Troubleshooting problem 2.
5. On the following day, carefully remove the media from the plate and replenish with 10 mL of pre-warmed D10 media.
6. To harvest retrovirus from transient viral production, filter the retrovirus-laden media from the transfected PlatE plate using a 0.45 μm syringe filter or a Steriflip filtration system, 48 h post-transfection.

***Note:*** It's best to use the freshly harvested retrovirus immediately for T cell transduction, though it can be stored at −80°C if necessary.

7. A stable virus-packaging PlatE culture can be prepared instead for a high-titer and stable viral production if the transduction efficiency or virus title is less than ideal:
  a. To enhance the integrated viral genome copy number in PlatE cells as well as their transfected percentage, perform a serial transfection by repeating Steps 2 to 5 two to three times.
  b. A day after the final transfection, culture the PlatE cells in D10 media supplemented with 10 μg/mL blasticidin and 1 μg/mL puromycin to select for gag-pol^+^ and env^+^ cells.
  c. Regularly monitor the proportion of PlatE cells expressing the fluorescent reporter (mTagBFP2 in this context) using fluorescent microscopy or flow cytometry, ensuring it remains at or above 90%.
    ***Note:*** Transfected PlatE cells will also express the CAR in your vector, which can be validated by flow cytometry via staining of the CAR-specific antibody alongside the fluorescent reporter.
  d. If the percentage of cells expressing the fluorescent reporter declines over time, consider purifying the PlatE cells using fluorescence-activated cell sorting or monoclonalization to achieve ≥ 95% purity.
  e. Once purified, expand and cryopreserve the PlatE culture. This culture now serves as a stable source for continuous virus production.
  f. To harvest retrovirus, first thaw and culture the stable virus-producing PlatE cells specific to your construct. Three days prior to T cell transduction, seed approximately 5 × 10^6^ PlatE cells in a 10 cm TC-treated dish using standard D10 media without blasticidin or puromycin.
  g. On the next day, ensure the cells have reached 70–80% confluency. Then, carefully remove the media and replace it with 10 mL of pre-warmed D10 media.
  h. After 48 h, filter the retrovirus-laden media from the plate using a 0.45 μm syringe filter or a Steriflip filtration system. Ideally, use the freshly harvested retrovirus immediately for T cell transduction. Alternatively, it can also be stored at −80°C if needed.
    **CRITICAL:** Both transfected and stable packaging cells continuously produce and release infectious retrovirus particles. These can pose biohazard risks or lead to cross-contamination if aerosolized. Always exercise caution and assess the risk of cross-contamination based on the tropism of the virus.
    ***Note:*** A stable virus-packaging cell culture can only be established for regular retroviral constructs that are not self-inactivating.
    ***Note:*** Store the retrovirus-laden medium at −80°C for less two weeks. Avoid repeated freezing/thawing cycles.

### Isolation and activation of mouse CD3^+^ T cells

**Timing: 2 days**

This section outlines the procedure for isolating CD3^+^ T cells from mouse spleens. The subsequent *in vitro* stimulation is crucial because gamma-retroviruses can only transduce cells that are actively proliferating.

8. Determine the number of donor mice required for your experiment. Typically, a single donor mouse can provide enough cells to support 8 to 10 recipient mice, with each recipient receiving an injection of 5 × 10^6^ transduced CAR T cells.

**CRITICAL:** Prior to initiating experiments involving mice, be sure to obtain the necessary approvals from the relevant institutional regulatory authority overseeing animal research. All procedures involving animals in this study strictly followed the University of Pennsylvania's guidelines for animal care and use.

9. The day before the T cell isolation, coat non-treated 6-well plates with 1.5 mL PBS containing 5 μg/mL anti-mouse CD3 antibody and 5 μg/mL anti-mouse CD28 antibody for each well. Seal the plates with Parafilm to prevent evaporation and incubate at 4°C overnight (12–16 h).

***Note:*** Anticipate needing 2 to 3 coated wells for every mouse spleen you plan to isolate.

10. On the day of the T cell isolation, euthanize the appropriate number of donor mice following the animal euthanization guidelines of your institute. Expect 6–12 × 10^7^ total splenocytes per mouse. T cells comprise the 25–30% of splenocytes.
11. Spray the bodies of the mice with a 70% (vol %) ethanol solution, collect the spleens, and place them in 2 mL of MACS isolation buffer within a sterile 6 cm cell culture dish on ice.
12. Cut the spleens into pieces that are 1–2 mm in size.
13. Transfer the spleen fragments to a 70 μm sterile cell strainer positioned over a 50 mL conical tube. Make sure the strainer is pre-rinsed with MACS buffer and not dry during the process.
14. Using a sterile syringe plunger, gently press the spleen fragments against the cell strainer. Rinse the strainer with 20 mL of MACS buffer to collect all cells.
15. Spin down the contents of the 50 mL conical tube at 300 × *g* for 5 min.
16. Carefully remove the supernatant and resuspend the resulting cell pellet in 1 mL of MACS buffer per spleen.
17. Proceed to isolate CD3^+^ T cells from the single-cell suspension using the EasySep mouse T cell isolation kit:
  a. Mix 20 μL of FcR blocker with every 1 mL of single-cell suspension that contains 1 × 10^8^ cells.
  b. To one 15 mL conical tube, transfer ≤ 4 mL of the cell suspension.
  c. To each 1 mL of cell suspension, add 50 μL of Isolation Cocktail and mix by pipetting.
  d. Incubate at room temperature (20°C–22°C) for 10 min.
  e. Thoroughly vortex the RapidSpheres for 30 s to ensure even dispersion.
  f. Add 75 μL of RapidSpheres to each 1 mL of cell suspension and mix.
  g. Incubate the mixture at room temperature (20°C–22°C) for 2.5 min.
  h. Top up the 15 mL tubes with the MACS isolation buffer. If the content volume in one tube is < 4 mL, top up to 5 mL; if the volume is ≥ 4 mL, top up to 9 mL. Mix by pipetting.
  i. Place the uncapped tube into the magnet and let it sit for 2.5 min at room temperature (20°C–22°C).
  j. Holding the magnet, pour the enriched CD3^+^ T cells into a fresh 15 mL tube in a single, smooth motion.
  k. Count the isolated T cells with a hemocytometer. Expect 25–30% of the starting number of splenocytes, around 6–12 × 10^7^T cells from each euthanized mouse.
  l. Centrifuge the isolated T cell suspension at 300 × *g* for 5 min.
18. Discard the supernatant and resuspend the cell pellets in TCM, aiming for a concentration of 1.5 × 10^6^ cells per mL.
19. Empty the antibody solution from the 6-well plate prepared the previous day and rinse each well with PBS. Do not allow the wells to dry during the process.
20. Add 2–3 mL of the T cell-suspended TCM to the coated 6-well plate. Place the plate in an incubator set at 37°C with 5% CO2, 48 h prior to T cell transduction. Expect a 2-fold expansion in the number of T cells in response to the activation after 48 h.

**CRITICAL:** Regularly check the T cells during the stimulation phase. If the medium appears distinctly yellow, consider adding 1 mL of fresh TCM to each well. The presence of enlarged cells and cell clusters indicates successful cell activation and stimulation.

### T cell transduction

**Timing: 5 h**

**Figure 4:**
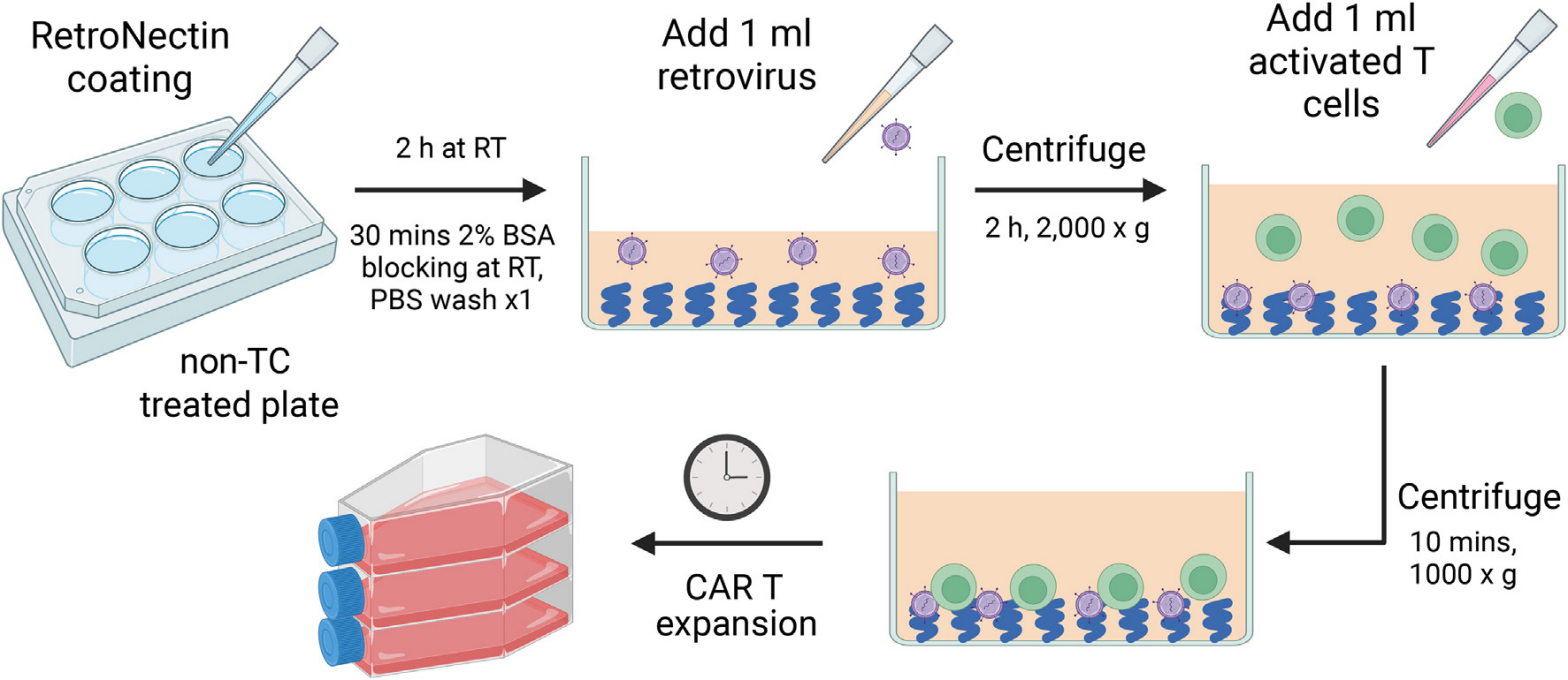
Schematic representation of the CAR T cell transduction A non-TC treated plate is initially coated with RetroNectin and allowed to incubate for 2 h at room temperature (RT, 20°C–22°C). This is followed by a 30-min 2% BSA blocking step at RT, and a subsequent wash with PBS. 1 mL of retrovirus is then added to the coated plate and centrifuged at 2,000 × *g* for 2 h. After centrifugation, 1 mL of activated T cells is introduced to the plate, after which the plate undergoes final centrifugation at 1,000 × *g* for 10 min. Transduced CAR T cells will be expanded for subsequent *in vitro* and *in vivo* assay.

This section outlines the procedure for transducing activated mouse T cells using an ecotropic gamma-retrovirus, facilitated by RetroNectin (Figure 4).

21. Prepare the RetroNectin-coated plates.
  a. Coat each well of a non-treated 12-well plate with 20 μg/mL RetroNectin solution in 0.75 mL PBS.
  b. Incubate the 12-well plate at room temperature (20°C–22°C) for 2 h.
  c. Remove the RetroNectin solution, and then block each well with 1 mL of 2% BSA in PBS. Avoid letting the wells dry out.
    ***Note:*** the RetroNectin solution could be stored at 4°C for up to one month and be recycled up to 3 times.
  d. Let the plate sit at room temperature (20°C–22°C) for an additional 30 min.
  e. Remove the BSA solution and replace it with 1 mL of PBS in each well. The plate is now primed for use.
22. If using frozen retrovirus stock, thaw it in a 37°C water bath. For fresh stock, pass the retrovirus supernatant through a 0.45 μm syringe filter or use a Steriflip filtration system.
23. Discard the PBS from the RetroNectin-coated plate.
24. Add 1 mL of retrovirus supernatant to each coated well. Ensure the wells remain moist. For control wells without the virus, use 1 mL of D10 medium.
25. Seal the 12-well plate with Parafilm to prevent virus supernatant aerosolization.
26. Spin the 12-well plate at 2,000 × *g* for 2 h, using the lowest or zero acceleration and brake settings. If you have a temperature-controlled centrifuge, set it to 32°C.
27. Take out the 12-well plates with T cells. Gently pipette the activated T cells in each well to detach them.
28. To break down cell clumps, pass the cell suspension through a 70 μm strainer.
29. Count the stimulated T cells with a hemocytometer.
30. Centrifuge stimulated T cells at 300 × *g* for 5 min.
31. Discard the supernatant and resuspend the T cells to a concentration of 3 × 10^6^ cells/mL in TCM.

***Note:*** You can set aside a sample of the T cells here as a non-transduced control.

32. To each well of the centrifuged 6-well plate, add 1 mL of the T cell suspension, ensuring a total volume of 2 mL per well.
33. Centrifuge the plate at 1,000 × *g* for 10 min, using the lowest or zero acceleration and brake settings. Troubleshooting problem 3.
34. Incubate the 12-well plate at 37°C with 5% CO2.
35. Maintain and expand the transduced CAR T cells.
  a. On the next day, homogenize and count the CAR T cells with trypan blue.
  b. If cell density exceeds 3 × 10^6^ cells/mL, bring it down to 1 × 10^6^ cells/mL with fresh TCM. Transfer the cells to a larger untreated vessel if necessary. Change the media entirely if the culture visually looks spent and yellow.
  c. Repeat Steps 35(a) and 35(b) every day for 2–4 days, aiming to expand the total living CAR-T cell count to at least 120% of the actual count required for all recipient mice.

***Note:*** For ideal T cell viability and efficacy, it is not recommended to expand the transduced CAR T cells *ex vivo* for more than 6 days.

### Assess CAR T cell transduction efficiency by flow cytometry

**Timing: 45 min**

Expression of the construct of interest can be validated by flow cytometry 48–72 h after the transduction. This section describes the steps of transduction efficiency assessment using the fluorescent reporter expression, as well as the staining of CAR expression.

**Figure 5:**
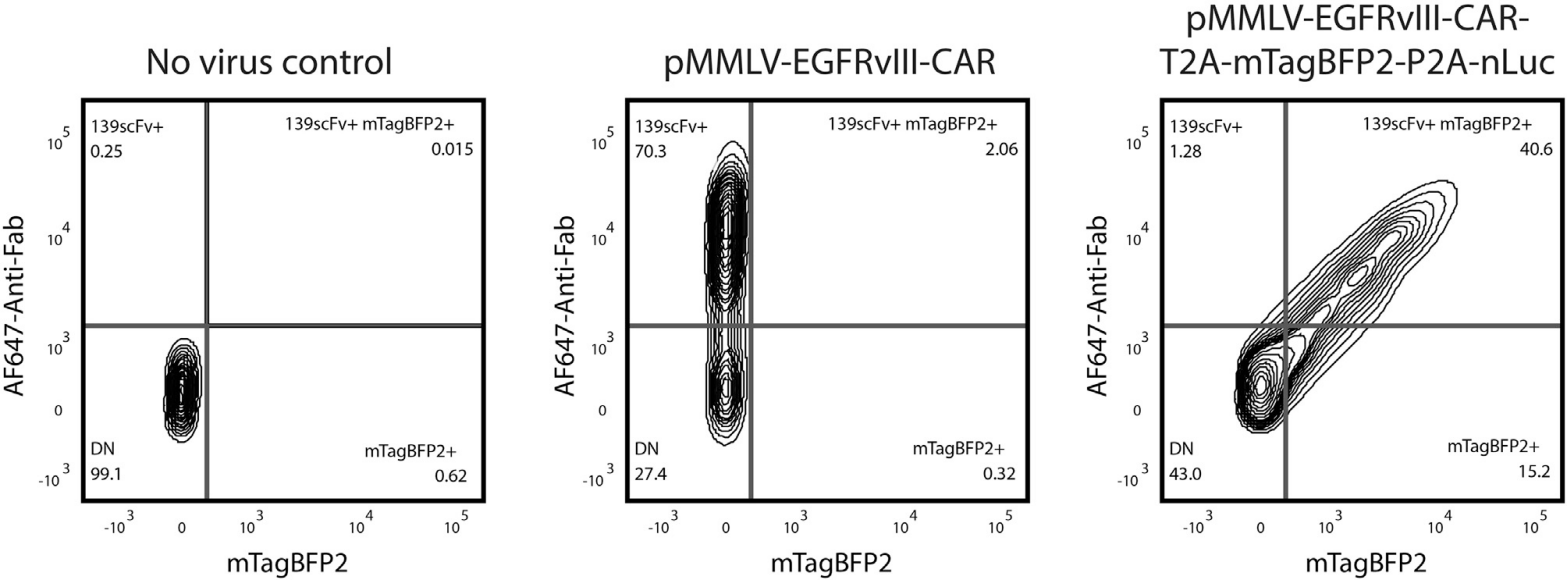
Flow cytometry analysis of T cells transduced with different constructs Density plots represent fluorescence intensity on a logarithmic scale for AF647-Anti-human scFv (y-axis) versus mTagBFP2 (x-axis). From left to right: (1) No-virus control; (2) CAR T cells transduced with pMMLV-139sFvCAR demonstrating specific CAR expression; (3) CAR T cells transduced with pMMLV-139sFvCAR-T2A-mTagBFP2-P2A-nLuc showcasing dual expression of 139scFvCAR and mTagBFP2. Percentage values indicate the proportion of cells within the designated quadrants.

36. Homogenize transduced CAR T cells as well as the no-virus control T cells; sample 100 μL of each into a 1.5 mL Eppendorf tube.
37. Add 900 μL PBS to each tube.
38. Vortex the samples briefly and centrifuge at 500 × *g* for 5 min.
39. Remove the supernatant and resuspend the cell pellets in 100 μL of MACS buffer, which contains the conjugated CAR-specific antibody at the correct concentration. For this protocol, we utilized an Alexa Fluor 647-conjugated goat anti-human Fab antibody at a 1:200 dilution.
40. Incubate the samples at room temperature (20°C–22°C) for 20 min on an orbital shaker set to ≤ 300 rpm. Ensure the samples are shielded from light during this period.
41. Add 900 μL PBS to each tube.
42. Vortex briefly and centrifuge again at 500 × *g* for 5 min.
43. Discard the supernatant and resuspend the cell pellets in 300 μL of MACS buffer.
44. Transfer each sample to a 5 mL round-bottom tube or other suitable flow cytometry tubes.
45. Add 5 μL of a 10 μg/mL propidium iodide (PI) solution in PBS to each sample and mix by pipetting. Immediately proceed to analyze the samples using a flow cytometer.
46. Evaluate the transduction efficiency from the acquired flow cytometry data.
  a. Gate out debris using the FSC-A / SSC-A plane.
  b. Remove non-singlet events by gating on the SSC-A / SSC-H plane.
  c. Exclude dead cells based on PI staining.
  d. Determine the transduction efficiency by calculating the percentage of mTagBFP2^+^/Egfrviii-CAR^+^ double-positive cells (Figure 5). The typically expected transduction efficiency is 30%–50%. Troubleshooting problem 4.

### Adoptive transfer of mouse CAR T cell

**Timing: 2–4 days**

This section outlines the introduction of transduced mouse CAR T cells into recipient mice.

47. Homogenize the expanded CAR T cells by pipetting. Centrifuge the cell suspension at 300 × *g* for 5 min.
48. Discard the supernatant and resuspend the cell pellet in PBS.
49. Count the cells with a hemocytometer.
50. Centrifuge the cell suspension at 300 × *g* for 5 min.
51. Remove the supernatant and reconstitute the cell pellet in PBS to achieve a concentration of 5 × 10^7^ cells per mL. This concentration allows for a dosage of 5 × 10^6^ cells in 100 μL for each mouse. Maintain the cell suspension on ice.
52. Administer 100 μL (equivalent to 5 × 10^6^ cells) of the cell suspension into each recipient mouse intravenously using a 31-gauge insulin syringe.

### Luciferase-based CAR T cell cytotoxicity assay

**Timing: 1.5 days**

This section outlines a luciferase-based CAR T cell cytotoxicity assay suitable for tumor cell lines expressing the CAR target and stably expressing firefly luciferase (fLuc). The viability of target tumor cells, when co-cultured with CAR-T effector cells, is directly assessed using a bioluminescence plate reader after introducing the firefly luciferase substrate, D-luciferin.

53. Prepare a single-cell suspension of the target tumor cells.
  a. Trypsinize the Egfrviii^+^ GL261 cells and resuspend them in D10 medium.
  b. Count the cells with a hemocytometer.
  c. Adjust the cell concentration based on the desired number of target cells per well. For instance, for 5,000 target cells in each well of a 96-well plate, set the concentration to 1 × 10^5^ cells/mL.
54. Prepare CAR T and control CAR T cell suspensions.
  a. Homogenize and resuspend CAR T and control CAR T cells in TCM.
  b. Count the cells with a hemocytometer.
  c. Adjust the T cell concentration to be 10 times that of the target cell concentration (e.g., 1 × 10^6^ T cells/mL for 1 × 10^5^ target cells/mL).
  d. Using TCM, dilute the T cells to prepare suspensions for each target-to-effector ratio group according to the following table:

**Table:** 

T:E ratio	10× T cell suspension	TCM	Total volume (for 4 wells)
1:10	200 μL	0 μL	200 μL
1:5	100 μL	100 μL	
1:3	60 μL	140 μL	
1:2	40 μL	160 μL	
1:1	20 μL	180 μL	
1:0.5	10 μL	190 μL

**Figure 6:**
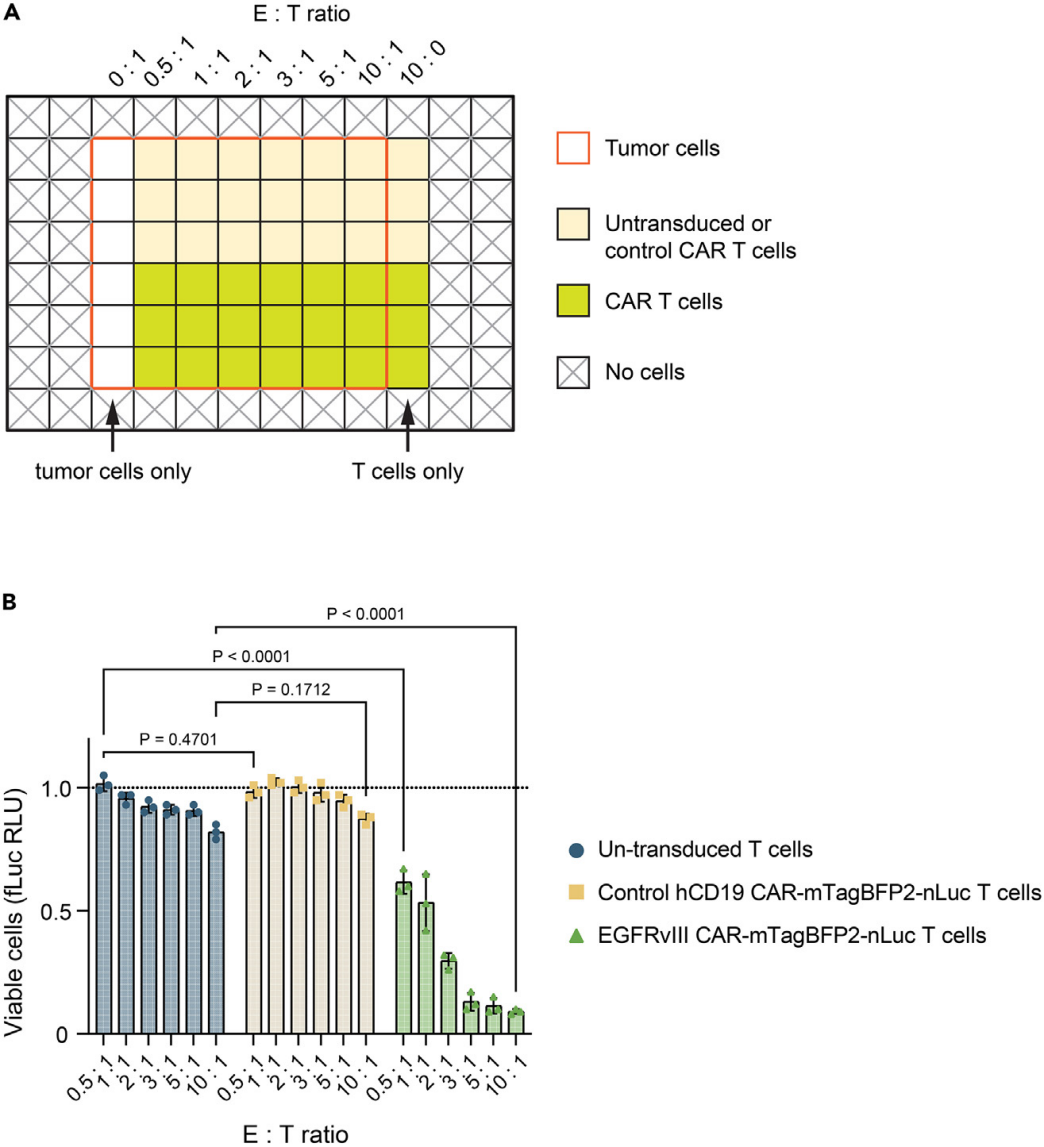
CAR T cytotoxicity assay set up and analyzed readout (A) Co-culture setup of CAR T cells and tumor cells. The red outline represents wells with tumor cells, the yellow squares represent the wells with non-transduced T cells or control CAR T cells, and the green squares represent the wells with CAR T cells. The varying T cell-to-tumor cell ratios are indicated along the top axis from a T cell-only condition (10:1) to a tumor cell-only condition (0:1). (B) Luciferase activity measurement, shown in Relative Light Units (RLU), normalized to no T cell (tumor cells only) wells. Different E:T (effector: target) ratios are plotted on the x-axis (mean ± SEM, from 3 technical replicates). Analysis by two-way ANOVA.

55. In a 96-well white-wall clear-bottom plate, seed both the target cells and T cells (Figure 6A). Each well should contain 100 μL: 50 μL of target cell suspension and 50 μL of T cell suspension. Ensure each target-to-effector combination is done in triplicate.
56. Incubate at 37°C in 5% CO_2_ for 24 h.
57. Add 10 μL of D-luciferin solution in PBS to each well being tested.
58. Shake the plate on an orbital shaker for 2 min.
59. Read the 96-well plate on a plate reader for the bioluminescence readout (Figure 6B).

### Dual-luciferase *in vivo* imaging for tumor and CAR T cells

**Timing: 1 h**

**Figure 7:**
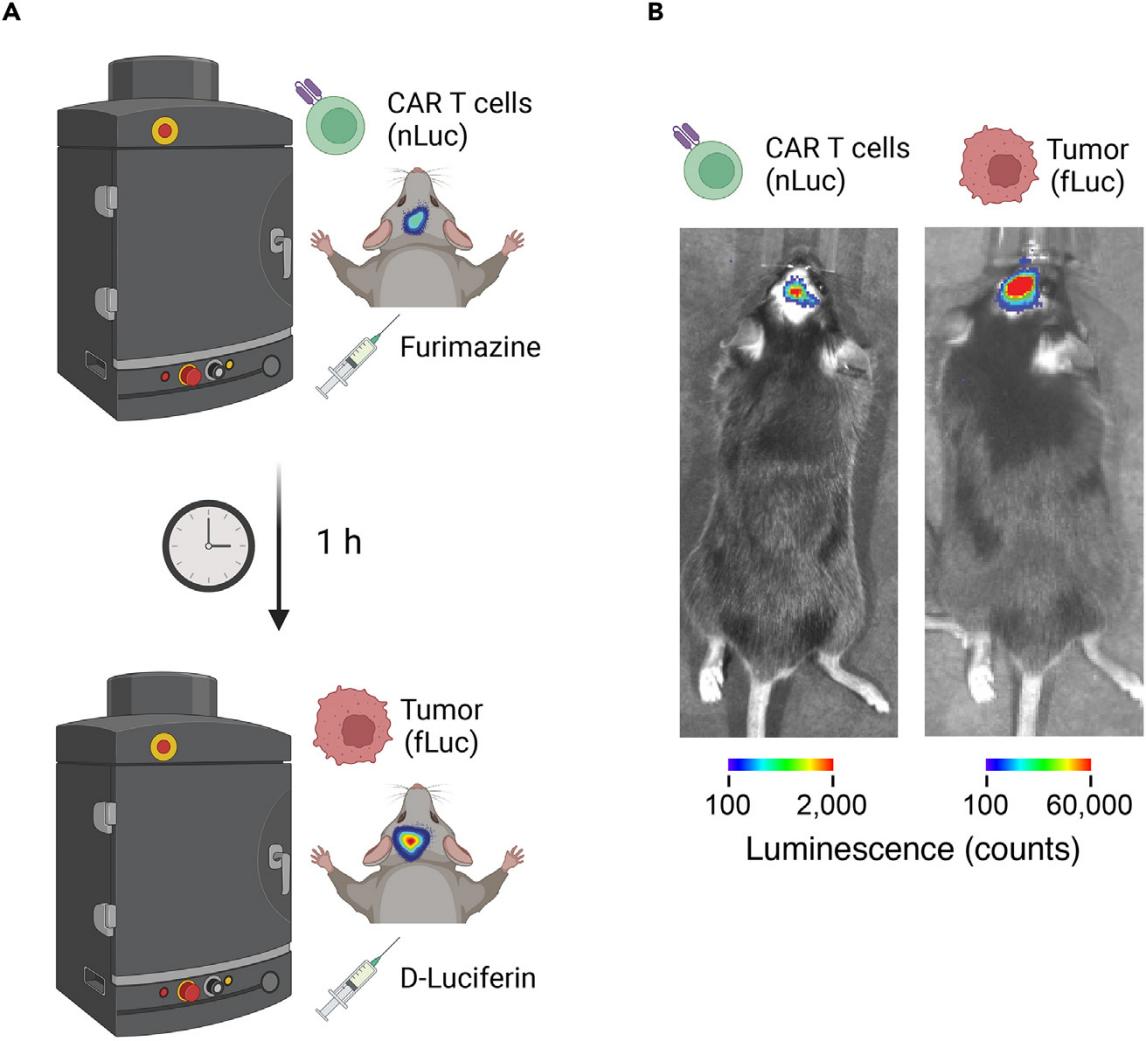
*In vivo* bioluminescence imaging of CAR T cells and tumors in mice (A) Schematic representation of the imaging procedure. Mice injected with CAR T cells expressing nLuc are placed inside the imaging chamber of an IVIS. Furimazine substrate is administered, and after an 1-h waiting period, the mice are again placed inside the imaging chamber, this time having tumors imaged by fLuc. Subsequent administration of D-Luciferin allows for the detection of fLuc-expressing tumors. (B) Representative bioluminescence images of mice. The left panel shows the distribution of CAR T cells (nLuc) post furimazine injection. The right panel displays the tumor (fLuc) distribution post-D-Luciferin injection.

This section outlines the procedure for bioluminescence imaging of GBM-bearing mice treated with the traceable CAR T cells using an IVIS (Figure 7A).

60. Determine the best timing for your *in vivo* imaging sessions. The initial infiltration of traceable CAR T cells can be observed as early as 1–2 days post-adoptive transfer and can be repeated every two days for weeks.
61. Calibrate and warm up the IVIS imaging system according to the manufacturer’s instructions.
62. Set the optimal parameters (exposure time, binning, FOV, and F/stop) for nano-luciferase imaging on the IVIS software.
63. Anesthetize the mice using an isoflurane/oxygen mixture. Start with 4% isoflurane for induction and then maintain anesthesia with 1–3% isoflurane.
64. Remove the fur on top of the head of your recipient mice using a trimmer or hair removal cream.
65. Dilute 5 μL of the furimazine 20× stock solution 20-fold in sterile saline or PBS (100 μL /mouse in total) for injection.
66. Immediately inject 100 μL of the 1× furimazine solution into the mice retro-orbitally with a 31-gauge insulin syringe.
67. Wait for 1 min to allow substrate distribution and reaction.
68. Place the anesthetized mice in the prone position on the imaging platform inside the IVIS chamber.

***Note:*** Ensure that the mice are evenly spaced and not overlapping. Make sure the flow of the isoflurane/oxygen mixture is allowed in the imaging chamber, since the mice need to remain anesthetized during the whole process.

69. Close the IVIS chamber to ensure darkness and start the imaging acquisition.
70. Once imaging is complete, remove the mice from the IVIS chamber. Monitor the mice until they fully recover from anesthesia.
71. Wait for ≥ 60 min to allow the nano-luciferase signal to wean off.
72. Set the optimal parameters (exposure time, binning, FOV, and F/stop) for firefly luciferase imaging on the IVIS software.
73. Anesthetize the mice using an isoflurane/oxygen mixture. Start with 4% isoflurane for induction and then maintain anesthesia with 1–3% isoflurane.
74. Inject 50 μL of the D-luciferin solution into the mice retro-orbitally with a 31-gauge insulin syringe. Avoid using the same eye you have used for the furimazine solution injection.
75. Wait for 1 min to allow substrate distribution and reaction.
76. Place the anesthetized mice in the prone position on the imaging platform inside the IVIS chamber. Ensure that the mice are evenly spaced and not overlapping.
77. Close the IVIS chamber to ensure darkness and start the imaging acquisition. Troubleshooting problem 5.
78. Once imaging is complete, remove the mice from the IVIS chamber. Monitor the mice until they fully recover from anesthesia.
79. Return the mice to their home cages and monitor for any adverse reactions, especially at the injection site.
80. Save the raw and analyzed images in a secure location. Document the imaging parameters, mouse details, and any observations made during the imaging session (Figure 7B).

## Expected outcomes

Upon successful completion of the protocol described in the document, researchers can anticipate the generation of mouse CAR T cells that are equipped with a CAR under a nano-luciferase and fluorescent protein-expressing tracing system. This unique system, facilitated by an MMLV retroviral vector, will enable the tracking of CAR T cell infiltration and presence *in vivo* within syngeneic mouse cancer models using a bioluminescence-based *in vivo* imaging system.

Upon isolating and expanding mouse spleen-derived T cells, one can expect a successful enrichment of CD3^+^ T cells from the single-cell suspension. The subsequent stimulation process should result in activated T cells ready for transduction.

When generating mouse CAR T cells using retrovirus, the transduction efficiency is a critical metric. After the transduction process, the expression of the construct of interest can be validated by flow cytometry 48–72 h post-transduction. Specifically, the flow cytometry data should reveal the transduction efficiency by assessing the fluorescent reporter expression and the staining of CAR expression. The cells should be gated to exclude debris, non-singlet events, and dead cells (using PI staining). The primary outcome to look for would be the percentage of mTagBFP2^+^/Egfrviii-CAR^+^ double-positive population, which indicates successful transduction (Figure 5).

The CAR system integrated into this protocol is specifically designed as a murine CAR construct containing 139 scFv. This construct targets Egfrviii, which is a GBM-associated epidermal growth factor receptor (EGFR) mutant. The luciferase-based *in vitro* cytotoxicity assay should confirm the successful induction of target cell death (Figure 6B). As a result, when the CAR T immunotherapy is administered to mice bearing Egfrviii-expressing syngeneic GL261 GBM tumors, one can expect a targeted response against these tumor cells.

Lastly, the protocol enables *in vivo* bioluminescence tracking of CAR T cells in mice. This is facilitated by the nano-luciferase component of the CAR construct, allowing researchers to trace CAR T cell infiltration and presence *in vivo* in syngeneic mouse cancer models using a bioluminescence-based *in vivo* imaging system. Successful execution should result in clear bioluminescent signals indicating the presence and distribution of CAR T cells within the mouse model (Figure 7B).

The protocol’s versatility suggests that it might be adaptable to other CAR-antigen combinations, different animal models, or alternative transgene carrier vectors. However, it’s essential to note that these potential applications have not been directly explored or optimized by the authors. Therefore, while the primary outcome remains the effective generation and tracking of CAR T cells in the described model, there lies the potential for broader applications and outcomes based on the foundational principles of this protocol.

## Quantification and statistical analysis

Statistical analysis was conducted with GraphPad Prism 9. The statistical significance of CAR T cell cytotoxicity was assessed using two-way ANOVA with Dunnett’s multiple comparison tests. Flow cytometry data were analyzed and visualized using FlowJo v10. Mouse IVIS images were processed and visualized with Living Image v4.8.

## Limitations

The transduction process relies on the use of an MMLV retroviral vector. The efficiency of retroviral transduction can vary, and there might be instances where the transduction efficiency is suboptimal. While flow cytometry can provide insights into the transduction efficiency, it might not capture the full functional capacity of the transduced cells, especially in an *in vivo* setting.

The *in vivo* bioluminescence-based imaging system used to track the infiltration and presence of traceable CAR T cells in GBM-bearing mice provides a non-invasive method to monitor CAR T cell dynamics. However, bioluminescence imaging has its limitations, including potential signal attenuation due to tissue depth or the presence of pigments, which might affect the accuracy of the readout.

Additionally, the protocol is designed for syngeneic mouse glioma models. This means that the findings and outcomes might not directly translate to other animal models or human clinical settings. The tumor microenvironment, immune responses, and other factors can vary significantly between species and even between different strains of mice.

## Troubleshooting

### Problem 1

Inefficient virus production by PlatE cells (Steps 1–3).

### Potential solution

• *PlatE cells not maintained under selection pressure.* Maintain continuous selection pressure on PlatE cells with blasticidin and puromycin to ensure the presence of gag-pol and env genes necessary for virus production. Loss of these genes can result in a drop in virus titer.
• *Suboptimal seeding density affecting cell health and transfection efficiency.* Seed PlatE cells at the correct density to ensure they are neither too sparse nor too confluent at the time of transfection. An optimal density allows for sufficient cell growth and division, which is crucial for high virus production.

### Problem 2

Low transfection efficiency in PlatE cells (Step 4).

### Potential solution

• *Suboptimal cell confluency at the time of transfection.* Monitor the PlatE cells to ensure they reach the optimal confluency of 70–80% before transfection. Overconfluent or sparse cells can lead to reduced transfection efficiency.
• *Ineffective transfection reagent or protocol.* Use a proven transfection reagent like Lipofectamine 3000 and follow the manufacturer’s instructions precisely. Ensure that the DNA-Lipofectamine complexes are formed properly and that the cells are incubated with these complexes under the right conditions.

### Problem 3

Inadequate retrovirus transduction efficiency (Steps 21–33).

### Potential solution

• *Retrovirus supernatant is not potent enough.* To ensure the retrovirus supernatant has a high titer, it is crucial to verify the viral titer before transduction. If the titer is low, consider concentrating the virus or producing a fresh batch to increase the potency.
• *The RetroNectin-coated plate is not prepared correctly.* For the RetroNectin-coated plate, ensure that the concentration of RetroNectin is accurate and that the plate is incubated at the correct temperature and duration as specified in the protocol. Any deviation can affect the coating efficiency and subsequent virus binding.
• *The centrifugation step is not performed under optimal conditions.* During centrifugation, ensure that the settings (speed, temperature, acceleration, and brake) are as recommended. Deviations can lead to suboptimal transduction.

### Problem 4

Inaccurate assessment of transduction efficiency (Steps 39–46).

### Potential solution

• *Inappropriate gating strategy in flow cytometry.* Develop a robust gating strategy for flow cytometry analysis. This includes proper gating out of debris and doublets, which can otherwise skew the results. Use forward and side scatter properties to exclude debris and apply singlet gating to ensure accurate analysis.
• *Inadequate exclusion of dead cells.* Use a viability dye such as propidium iodide effectively to exclude dead cells from the analysis. Dead cells can bind non-specifically to antibodies and give false positives; hence it is crucial to exclude them accurately.
• *Suboptimal staining protocol.* Follow the staining protocol carefully, ensuring that the cells are incubated with the fluorescent antibodies under the right conditions. If the staining is suboptimal, consider optimizing the antibody concentration or the incubation time and temperature.

### Problem 5

Inaccurate *in vivo* imaging (Steps 61–77).

### Potential solution

• *Bioluminescence signal is weak or obscured.* If the bioluminescence signal is weak, ensure that the substrate (furimazine) is fresh and properly diluted. Also, check the imaging equipment settings and adjust the exposure time, binning, and F/stop to optimize signal detection.
• *Anesthesia is not properly maintained during imaging.* Properly maintain anesthesia by monitoring the mice closely and adjusting the isoflurane levels as needed to keep the mice sedated but stable throughout the imaging procedure.
• *Furimazine substrate is not prepared or administered correctly.* Prepare the furimazine substrate immediately before injection to ensure its potency. Administer it carefully and consistently to each mouse to ensure reliable and comparable imaging results.

***Alternatives:*** The solubility problem for furimazine can be circumvented by using hydrofurimazine as an alternative, which has better solubility in aqueous solution and also a higher bioavailability.^9^

## Resource availability

### Lead contact

Further information and requests for resources and reagents should be directed to and will be fulfilled by the lead contact, Yi Fan, fanyi@upenn.edu.

### Technical contact

Duo Zhang, duozhang@pennmedicine.upenn.edu.

### Materials availability

This study did not generate new unique reagents.

### Data and code availability

This study did not generate new datasets or code.
